# Characteristics of Very Low Frequency Sound Propagation in Full Waveguides of Shallow Water

**DOI:** 10.3390/s21010192

**Published:** 2020-12-30

**Authors:** Nansong Li, Hanhao Zhu, Xiaohan Wang, Rui Xiao, Yangyang Xue, Guangxue Zheng

**Affiliations:** 1 Acoustic Science and Technology Laboratory, Harbin Engineering University, Harbin 150001, China; linansong@hrbeu.edu.cn (N.L.); zgx1057@hrbeu.edu.cn (G.Z.); 2 Key Laboratory of Marine Information Acquisition and Security (Harbin Engineering University), Ministry of Industry and Information Technology, Harbin 150001, China; 3 College of Underwater Acoustic Engineering, Harbin Engineering University, Harbin 150001, China; 4 Institute of Marine Science and Technology, Zhejiang Ocean University, Zhoushan 316022, China; zhuhanhao@zjou.edu.cn; 5 State Key Laboratory of Acoustics, Chinese Academy of Sciences, Beijing 100190, China; 6 Institute of Naval Architecture and Mechanical-Electrical Engineering, Zhejiang Ocean University, Zhoushan 316022, China; S18082400018@zjou.edu.cn (R.X.); S19082400018@zjou.edu.cn (Y.X.)

**Keywords:** very low frequency (VLF), sound propagation characteristic, full waveguides, finite element method (FEM), fluid/elastic interaction

## Abstract

This work is concerned with the characteristics of very low frequency sound propagation (VLF, ≤100 Hz) in the shallow marine environment. Under these conditions, the classical hypothesis of considering the sea bottom as a fluid environment is no longer appropriate, and the sound propagation characteristics at the sea bottom should be also considered. Hence, based on the finite element method (FEM), and setting the sea bottom as an elastic medium, a proposed model which unifies the sea water and sea bottom is established, and the propagation characteristics in full waveguides of shallow water can be synchronously discussed. Using this model, the effects of the sea bottom topography and the various geoacoustic parameters on VLF sound propagation and its corresponding mechanisms are investigated through numerical examples and acoustic theory. The simulation results demonstrate the adaptability of the proposed model to complex shallow water waveguides and the accuracy of the calculated acoustic field. For the sea bottom topography, the greater the inclination angle of an up-sloping sea bottom, the stronger the leak of acoustic energy to the sea bottom, and the more rapid the attenuation of the acoustic energy in sea water. The effect of a down-sloping sea bottom on acoustic energy is the opposite. Moreover, the greater the pressure wave (P-wave) speed in the sea bottom, the more acoustic energy remains in the water rather than leaking into the bottom; the influence laws of the density and the shear wave (S-wave) speed in the sea bottom are opposite.

## 1. Introduction

Sound propagation in shallow water has always been a hotspot in the field of underwater acoustics, and is the basis for understanding, predicting, and applying various shallow sea acoustic phenomena [1]. Due to the development of submarine stealth technology and the requirements for monitoring various physical ocean phenomena, the detection methods for targets in shallow water gradually turn to very low frequency (VLF, ≤100 Hz); this is closely related to the propagation characteristics of VLF acoustic signals in shallow water. Therefore, the research on low-frequency sound propagation in shallow water has received increasing attention. The oceans surrounding China are mostly shallow and have a typical shallow water waveguide environment [2,3]. As a result, in recent years VLF sound propagation in shallow water has received more considerable attention within the acoustic modeling community in China [4]. Nevertheless, the existing research on sound propagation in shallow water uses horizontal layered waveguides for the environment models and treats the sea bottom as a range-independent fluid medium [5]. Research on modeling of VLF acoustic fields in shallow water with complex terrain changes that treats the sea bottom as an elastic medium is scarce [6]. Actually, the sea bottom structures in shallow water are composed of a sedimentary layer of a near-porous medium and a basement layer of a near-elastic medium [7]. Because of their long wavelength and strong penetrating power, the VLF underwater acoustic signals would penetrate both the sedimentary layer and the basement layer when propagating in shallow water. The sea bottom should therefore be considered as a range-dependent elastic medium when studying the characteristics of VLF sound propagation in shallow water.

In this type of sea environment model, the strong penetrability of VLF acoustic signals could cause substantial acoustic energy to leak into the sea bottom, generating seismic acoustic signals that propagate through the sea bottom or bottom surface [8,9,10]. These seismic acoustic signals generally attenuate more slowly than acoustic signals propagating through sea water, thus they can be detected and identified at longer distances [9] and have broad applications in detection for any targets in shallow water [11,12,13]. But the existing research has mostly focused on the modeling and distribution of the propagation characteristics of VLF acoustic signals in only the sea water layer. Due to the lack of research findings, there are still few kinds of sensors or equipment that have been developed for any applications with the seismic acoustic signals. Therefore, when discussing the characteristics of VLF sound propagation in shallow water, it is important to comprehensively analyze the propagation characteristics in a full waveguide model which unifies the sea water and sea bottom in shallow water.

Existing research on sound propagation is based on reasonable acoustic field calculation methods. Over the years, a number of acoustic field calculation methods, such as the normal mode method, ray method, parabolic equation method, fast field method, and their derivatives, have been established to study sound propagation in shallow water. These methods are based on limiting assumptions and approximations of the wave equation and sea environment, restricting their universality, especially for the calculation of sound propagation at VLF in range-dependent shallow water with an elastic bottom [14]. As increasing attention is being paid to VLF sound propagation in range-dependent waveguides, it is critical to establish a shallow water acoustic field calculation method with greater universality. The finite element method (FEM) accurately describes the changes in an acoustic field by dividing the environment into discrete units [15]. In the past, beyond providing reference solutions, the FEM has seldom been used for sound propagation in the sea due to the large calculations involved. However, due to advances in computer technology, it is now possible to implement the FEM for sound propagation in shallow water.

To improve the existing research on the propagation characteristics of VLF acoustic signals in shallow water, in this paper we propose a finite element calculation method for VLF sound propagation. Taking the acoustic energy flux as the research object [16], we discuss the propagation characteristics of VLF acoustic signals in range-dependent full waveguides of shallow water with an elastic bottom. This paper is organized as follows: a calculation method for VLF underwater acoustic field based on FEM is described in Section 2.1, and the formulas for the acoustic energy flux in sea water and sea bottom of shallow water are given in Section 2.2. In Section 3, the effects of the sea bottom topography and the geoacoustic parameters on VLF sound propagation and its corresponding mechanisms are elaborated with numerical examples. Finally, our conclusions are presented in Section 4.

## 2. Theoretical Model

### 2.1. Finite Element Form of Acoustic Field in Shallow Water

**Figure 1:**
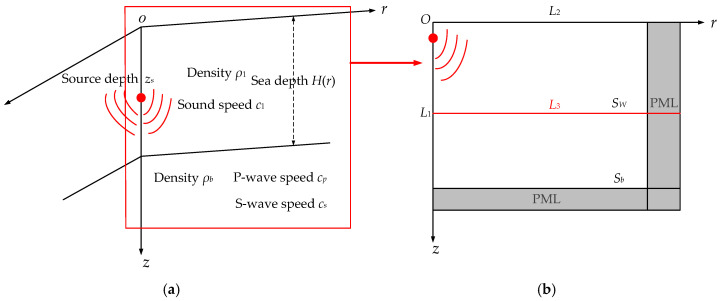
The full waveguide model unifies the sea water and the sea bottom in shallow water. (**a**) The waveguide model in three-dimensional cylindrical coordinates; (**b**) the solution region in the (*r*, *z*) plane.

To explore the characteristics of VLF sound propagation in shallow water, a waveguide model that unifies the sea water and the sea bottom is established in three-dimensional cylindrical coordinates (*r*, *θ*, *z*), and the *N* × 2*D* hypothesis is adopted for this model. Under this hypothesis, the acoustic wave coupling between adjacent two-dimensional vertical planes (*r*, *z*) in the *θ* direction is neglected, and the original three-dimensional solution region is solved into a two-dimensional region in the (*r*, *z*) plane. Schematic diagrams of the full waveguide researched here are shown in Figure 1, with the model in three-dimensional cylindrical coordinates and the solution region in the (*r*, *z*) plane shown in Figure 1a,b, respectively.

In the solution region, the waveguide is regarded as a range-dependent environment that presents a uniform fluid medium, *S_w_*, above an infinite uniform elastic half space, *S_b_*, and the sea depth *H*(*r*) depends on the horizontal range, *r*. The sea surface is defined by the plane *z* = 0, and the *z-*axis indicates the depth of this region. The *r*-axis represents the direction of horizontal propagation of the acoustic signals. *ρ*_1_ and *c*_1_ are the density and the sound speed, respectively, in sea water. *ρ_b_*, *c_p_*, and *c_s_* are the density, pressure wave (P-wave) speed, and shear wave (S-wave) speed, respectively, in the sea bottom [6], and *z_s_* is the depth of the point acoustic source which is set on the symmetric axis of the cylindrical coordinate, *L*_1_. *L*_2_ is the air/fluid interaction boundary between the air and the sea water, while *L*_3_ is the fluid/elastic interaction boundary between the sea water and the sea bottom.

The FEM is based on the weak form equation [16,17], which discretizes the calculated models to finite elements and then calculates the solution of all the elements. The approximate solution of the model in each element can be obtained according to the variable value of the element node.

By combining the weighted integration of the Helmholtz equation with Gaussian theory [18], the finite element equation for *S_w_* and *S_b_* in Figure 1 can be expressed as

(1) $$\left(K_{w}+i\omega C_{w}-\omega^{2}M_{w}\right)\left\{p_{i}\right\}=\left\{F_{wi}\right\}$$

(2) $$\left(K_{b}+i\omega C_{b}-\omega^{2}M_{b}\right)\left\{s_{i}\right\}=\left\{F_{bi}\right\}$$

In Equation (1), *p_i_* is the sound pressure at the *i*th node in the sea water layer, **M***_w_*, **K***_w_*, and **C***_w_* are respectively the mass matrix, the stiffness matrix, and the damping matrix in the fluid medium, {**F***_wi_*} is the acoustic excitation at this node. In Equation (2), *s_i_* is the displacement at the *i*th node in the sea bottom, **M***_b_*, **K***_b_*, and **C***_b_* are respectively the mass matrix, the stiffness matrix, and the damping matrix in elastic medium without boundary constraints, and {F*_bi_*} is the excitation load at this node.

When calculating the air/fluid interaction boundary, the *L*_2_ is set as a Dirichlet boundary, as defined in Equation (3).

(3) $$\begin{array}{cc} p(r,z)=0 & \left(\mathrm{on}\;L_{1}\right) \end{array}$$

*L*_3_ is set as a fluid/elastic coupling boundary, which should satisfy coupling conditions such as continuous normal displacement, continuous normal stress, and zero tangential stress. The definitions are as follows:

(4) $$\begin{array}{cc} \frac{\partial (\lambda_{w}\Delta_{w})}{\partial z}+\rho_{1}\omega^{2}s_{b}^{z}=0 & \left(\mathrm{on}\;L_{3}\right) \end{array}$$

(5) $$\lambda_{w}\Delta_{w}=\begin{array}{cc} \lambda_{b}s_{b}^{r}+\left(\lambda_{b}+2\mu_{b}\right)\frac{\partial s_{b}^{z}}{\partial z} & \left(\mathrm{on}\;L_{3}\right) \end{array}$$

(6) $$\frac{\partial \left(\lambda_{b}s_{b}^{r}\right)}{\partial z}\begin{array}{cc} +\frac{\partial }{\partial z}\left[\left(\lambda_{b}+2\mu_{b}\right)\frac{\partial s_{b}^{z}}{\partial z}\right]+\rho_{b}\omega^{2}s_{b}^{z}=0 & \left(\mathrm{on}\;L_{3}\right) \end{array}$$

$$\Delta =\frac{\partial s_{n}^{r}}{\partial r}+\frac{\partial s_{n}^{z}}{\partial z},n=w,b$$

where *s_n_^r^* and *s_n_^z^* (*n* = *w*, *b*) represent the displacement along the *r*-axis and *z*-axis in medium *S_w_* and medium *S_b_*. *λ_n_* and *μ_n_* (*n* = *w*, *b*) respectively represent the Lame constant in medium *S_w_* and medium *S_b_*. To simulate sound propagation in an infinite sea environment, a perfectly matched layer (PML) is used for the boundary treatment, as shown in Figure 1 [16].

Based on the FEM, by coupling the Helmholtz equation, fluid-elastic coupling boundary, and PML boundary, the acoustic pressure field *p* in fluid medium *S_w_* and the displacement field **s** in elastic half space *S_b_* can be calculated.

### 2.2. Acoustic Energy Flux in Full Waveguide of Shallow Water

In previous studies on the characteristics of acoustic intensity in a shallow water waveguide, the study object was the pressure field *p* [3,5]. However, *p* only reflects the propagation characteristics of the P-wave in a fluid medium. Because the sea bottom is considered as an elastic medium in this paper, it is impossible to correctly describe the propagation characteristics of acoustic intensity in a full waveguide of shallow water with *p*. Unlike *p*, the acoustic energy flux ***I*** exists in both the fluid medium and the elastic medium, making it more suitable for revealing the propagation characteristics of acoustic intensity in the full waveguide. Therefore, in this paper, the propagation characteristics of VLF acoustic intensity in a shallow water waveguide is discussed in terms of the acoustic energy flux.

In the time domain, the acoustic energy flux ***I*** is the time average of the instantaneous acoustic intensity ***I***(*t*). For an isotropic elastic medium, its definition is [19,20]:

(7) $$I(t)=\left[T\left(t\right)\cdot v\left(t\right)\right]=\left[\begin{array}{c} I_{r}\left(t\right)\\ I_{\theta }\left(t\right)\\ I_{z}\left(t\right) \end{array}\right]$$

$$T\left(t\right)=\left[\begin{array}{ccc} T_{rr}\left(t\right) & T_{r\theta }\left(t\right) & T_{rz}\left(t\right)\\ T_{\theta r}\left(t\right) & T_{\theta \theta }\left(t\right) & T_{\theta z}\left(t\right)\\ T_{zr}\left(t\right) & T_{z\theta }\left(t\right) & T_{zz}\left(t\right) \end{array}\right]\;v\left(t\right)=\left[\begin{array}{c} v_{r}\left(t\right)\\ v_{\theta }\left(t\right)\\ v_{z}\left(t\right) \end{array}\right]$$

where ***T*** and ***v*** are respectively the stress matrix and particle velocity field in the medium under three-dimensional cylindrical coordinates; ***T*** and ***v*** can be obtained through the corresponding relationship between *p*, ***s*** [21]. As the acoustic field is independent to the azimuthal angle *θ* in the cylindrical coordinate, Equation (7) can be simplified as:

(8) $$I(t)=\left[\begin{array}{ccc} T_{rr}\left(t\right) & 0 & T_{rz}\left(t\right)\\ 0 & 0 & 0\\ T_{zr}\left(t\right) & 0 & T_{zz}\left(t\right) \end{array}\right]\cdot \left[\begin{array}{c} v_{r}\left(t\right)\\ 0\\ v_{z}\left(t\right) \end{array}\right]=\left[\begin{array}{c} I_{r}\left(t\right)\\ 0\\ I_{z}\left(t\right) \end{array}\right]$$

In the time domain, the acoustic energy flux ***I*** can be obtained by computing the time average of Equation (8). According to Parseval’s theorem, the average acoustic energy flux ***I***(*ω*) in the frequency domain can be expressed as:

(9) $$I(\omega )=\left[\begin{array}{c} I_{r}\left(\omega \right)\\ 0\\ I_{z}\left(\omega \right) \end{array}\right]=\left[\begin{array}{ccc} T_{rr}\left(\omega \right) & 0 & T_{rz}\left(\omega \right)\\ 0 & 0 & 0\\ T_{zr}\left(\omega \right) & 0 & T_{zz}\left(\omega \right) \end{array}\right]\cdot {\left[\begin{array}{c} v_{r}\left(\omega \right)\\ 0\\ v_{z}\left(\omega \right) \end{array}\right]}^{*}$$

where the asterisk (*) denotes the complex conjugate.

As the elements in the vector ***I***(*ω*) are complex, the real part Re[***I***(*ω*)] represents the acoustic energy that can propagate over long distances, while the imaginary part Im[***I***(*ω*)] represents the acoustic energy that does not propagate. When sound waves propagate through an elastic medium, the amplitude and direction of the complex vector ***I***(*ω*) are defined as follows:

(10) I={Re[Ir(ω)]}2+{Re[Iz(ω)]}2

(11) θI=arctan{Re[Iz(ω)]Re[Ir(ω)]}

Since *μ* = 0, *T_rz_* = *T_zr_* = 0 and *T_rr_* = *T_zz_* = *λ*∇^2^*φ_p_* = *p* in the fluid, the equation for the acoustic energy flux in the fluid layer can be written as:

(12) $$I(\omega )=\left[\begin{array}{c} I_{r}\left(\omega \right)\\ 0\\ I_{z}\left(\omega \right) \end{array}\right]=\left[\begin{array}{c} pv_{r}^{*}\\ 0\\ pv_{z}^{*} \end{array}\right]$$

Using Equations (9) and (12), the acoustic energy flux ***I***(*ω*) in each layer of the full waveguide in shallow water can be calculated. In the following sections, we consider ***I***(*ω*) as the research object for investigation of the propagation characteristics of VLF acoustic signals in shallow water.

## 3. Simulation and Discussion

In this section, using numerical examples, we first verify the accuracy of the FEM simulation results for VLF sound propagation in shallow water, and then elaborate the effects of the sea bottom topography and the geoacoustic parameters on the characteristics of VLF sound propagation and its corresponding mechanisms.

### 3.1. Comparison of Simulation Results

**Figure 2:**
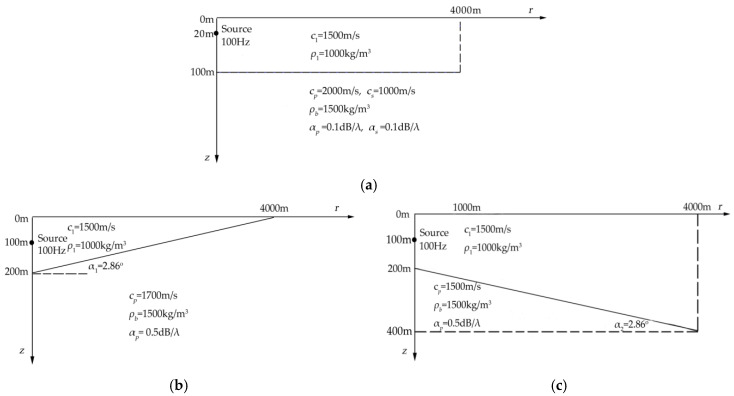
The simulation conditions and schematic diagrams of three types of shallow water models in comparison. (**a**) A shallow water environment with horizontal sea bottom; (**b**) a shallow water environment with wedge-shaped uphill sea bottom; the sea water depth is decreased linearly from 200 m at 0 km to 0 m at 4 km at a 2.86° slope angle; (**c**) a shallow water environment with wedge-shaped downhill sea bottom; the sea water depth increases linearly from 200 m at 0 km to 400 m at 4 km also at a 2.86° slope angle.

We verify the FEM simulation of the VLF underwater acoustic field for three types of sea bottom topography, horizontal [20], wedge-shaped uphill [14], and wedge-shaped downhill [22], by comparison with existing research results. The two-dimensional solution region and environmental parameters for the three types of shallow water environments are shown in cylindrical coordinates in Figure 2.

In each environment illustrated in Figure 2, the sound speed and density of the sea water are respectively set to *c*_1_ = 1500 m/s and *ρ*_1_ = 1000 kg/m^3^. Figure 2a depicts a horizontally layered model of a shallow water waveguide that treats the sea bottom as an elastic medium, in which the density, P-wave speed, and S-wave speed are respectively set to *ρ_b_* = 1500 kg/m^3^, *c_p_* = 2000 m/s (attenuation for P-wave is *α_p_* = 0.1 dB·λ^−1^), and *c_s_* = 1000 m/s (attenuation for S-wave is *α_s_* = 0.1 dB·λ^−1^), the depth of the 100 Hz point acoustic source is 20 m. In Figure 2b, the sea bottom for the shallow water is wedge-shaped uphill, as the ASA model [23,24], in which the sea water depth is decreased linearly from 200 m at 0 km to 0 m at 4 km, so the uphill slope angle is α_1_ ≈ 2.86°, the density and P-wave speed in this model are *ρ_b_* = 1500 kg/m^3^, *c_p_* = 1700 m/s (attenuation for P-wave is *α_p_* = 0.5 dB·λ^−1^), the depth of the 100 Hz point acoustic source is 100 m. For the downhill sea bottom model in Figure 2c, the sea water depth increases linearly from 200 m at 0 km to 400 m at 4 km at a slope angle of α_2_ ≈ 2.86°; the density, P-wave speed, and acoustic source are set the same as in Figure 2b.

**Figure 3:**
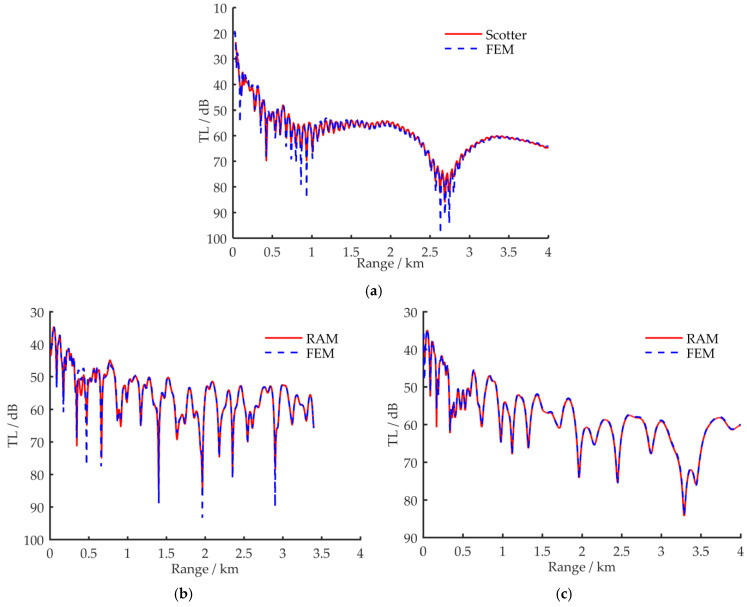
The comparison of the sound pressure Transmission Loss (TL) curves between FEM and other sound field simulation methods. (**a**) Comparison results between FEM and the fast field method under the simulation condition in Figure 2a; (**b**) comparison results between FEM and the coupled normal wave method under the simulation condition in Figure 2b; (**c**) comparison results between FEM and the parabolic equation method under the simulation condition in Figure 2c.

For the three simulation conditions, the sound pressure Transmission Loss (TL) curves calculated by FEM and other sound field simulation methods are shown for comparison in Figure 3. The receiving position is set at a depth of *z* = 30 m for all simulations. The sound pressure TL calculation formula is given by Equation (13):

(13) $$TL_{p}=-20\mathrm{lg}\left|\frac{p}{{p_{ref}|}_{r=1m}}\right|;\;p_{ref}=\frac{e^{ik_{0}r}}{r}$$

In Figure 3, the dotted lines are calculated results by FEM, and the solid lines are the results by three comparison acoustic field simulation methods. Based on their applicability, the fast field method (Scotter program) [17,25,26] and the parabolic equation method (RAM program) [26,27] are used to examine the error of FEM for the models in Figure 2a–c, respectively. The comparison results in Figure 3 show that the TL curves that were simulated by FEM are highly consistent with the results obtained by the existing simulation programs. This comparison verifies the accuracy of FEM for the simulation of underwater acoustic fields in shallow water. Considering the FEM is more suitable for the acoustic field simulation in complex ocean environments and can be used to solve the acoustic field in full waveguides of shallow water, the FEM is used for simulation of the characteristics of VLF sound propagation.

### 3.2. The Effects of Sea Bottom Topography on the Characteristics of VLF Sound Propagation

In this section, the effects of sea bottom topography on VLF sound propagation in full waveguides of shallow water are discussed. The two most common types of sea bottom topography, wedge-shaped uphill and wedge-shaped downhill, are discussed in Section 3.2.1 and Section 3.2.2, respectively.

#### 3.2.1. VLF Sound Propagation in Shallow Water with Wedge-Shaped Uphill Sea Bottom

**Figure 4:**
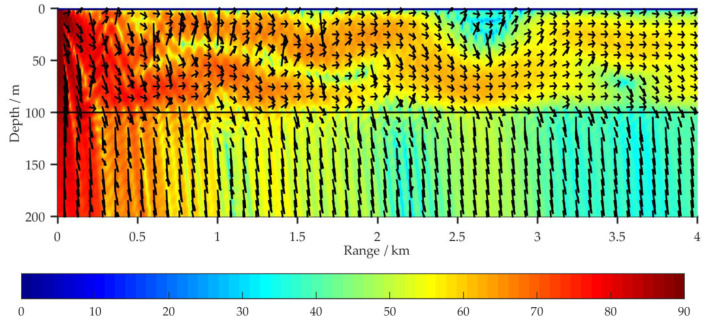
Distribution of acoustic energy flux (SIL) in full waveguide of shallow water for the simulation condition in Figure 2a with Δ*H*_1_ = 100 m.

**Figure 5:**
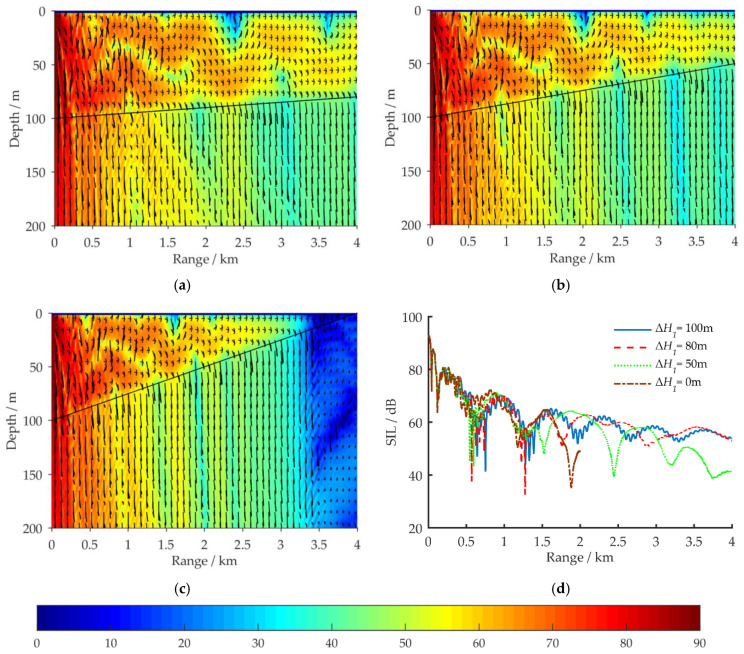
Distribution of the 100 Hz acoustic energy flux (SIL) in a full waveguide of shallow water with wedge-shaped uphill sea bottoms; aside from Δ*H*_1_, the environmental parameters are the same as in Figure 2a: (**a**) Δ*H*_1_ = 80 m; (**b**) Δ*H*_1_ = 50 m; (**c**) Δ*H*_1_ = 0 m; (**d**) comparison results of SIL curves for four simulation conditions: Δ*H*_1_ = 100, 80, 50, and 0 m, with a receiving depth of *z* = 50 m.

In Figure 4 and Figure 5, for shallow water environments with a horizontal sea bottom and a wedge-shaped uphill sea bottom, respectively, the propagation characteristics of the acoustic energy flux in the (*r*, *z*) region are compared for a VLF acoustic source at 100 Hz. The sound intensity level (SIL) for acoustic energy flux is defined by Equation (14):

(14) $$\mathrm{SIL}=10\mathrm{lg}\left|\frac{I}{I_{ref}}\right|;\;I_{\mathrm{ref}}=6.76\times 10^{-19}\;W/m^{2}$$

For the simulation, aside from the sea bottom topography, the environmental parameters have the same values as in Figure 2a. Δ*H*_1_ represents the vertical distance between the sea surface and the sea bottom at *r* = 4 km. The amplitude and direction of the acoustic energy flux at each grid point are represented by the length and direction of the arrows.

**Figure 6:**
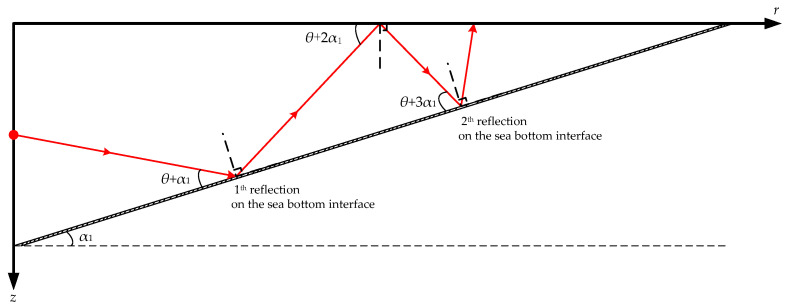
Distribution of the ray tracing when acoustic signals propagate in a shallow water environment with wedge-shaped uphill sea bottom, and the uphill slope angle of the wedge-shaped bottom is α_1_.

Figure 5 depicts the distribution of the 100 Hz acoustic energy flux in a full waveguide of shallow water for a wedge-shaped uphill sea bottom. Aside from Δ*H*_1_, the environmental parameters are the same as in Figure 2a. Figure 5a–c respectively depict the simulation results at Δ*H*_1_ = 80, 50, and 0 m with corresponding uphill slope angles of α_1_ = 0.29°, 0.72°, and 1.43°. Figure 5d shows a comparison of the propagation characteristics of the 100 Hz acoustic energy flux for four sea bottom simulation conditions: Δ*H*_1_ = 100, 80, 50, and 0 m. The receiving depth in the sea water is *z* = 50 m. Figure 6 depicts the distribution of the ray tracing when acoustic signals propagate in a shallow water environment with wedge-shaped uphill sea bottom, and the uphill slope angle of the wedge-shaped bottom is α_1_.

According to normal mode method, under the simulation conditions in Figure 2a, nine orders of normal modes would be excited in the shallow water waveguide at 100 Hz, and the grazing angles *θ* of these normal modes on the horizontal sea bottom interface are in the range [4°, 40°]. The higher the normal mode order is, the larger the grazing angle [17]. By comparing the simulation results in Figure 4 and Figure 5, it can be seen that under the influence of the uphill slope angle of the sea bottom *α*_1_, after its *n*th reflection on the sea bottom interface, the grazing angle of each excited normal mode is increased by (2*n* − 1)*α*_1_ degrees, as shown in Figure 6. For VLF sound propagation in shallow water with a wedge-shaped uphill sea bottom, the propagation characteristics of the low-order normal modes, which correspond to small grazing angles, are continuously coupled to the high-order normal modes, which correspond to large grazing angles, significantly strengthening the leakage effect of the acoustic energy to the sea bottom and relieving the fluctuation. The larger the slope angle α_1_ is, the greater the number of low-order normal modes that are coupled to higher orders, the more the acoustic energy leaks into the sea bottom, the faster the attenuation of the acoustic energy in the sea water, the fewer normal mode orders, and the simpler the normal mode interference in far-field regions. For these reasons, under the simulation conditions in Figure 6, as Δ*H*_1_ changes from 100 to 0 m, the 100 Hz acoustic energy flux reveals a loss of more than 20 dB.

#### 3.2.2. VLF Sound Propagation in Shallow Water with Wedge-shaped Downhill Sea Bottom

**Figure 7:**
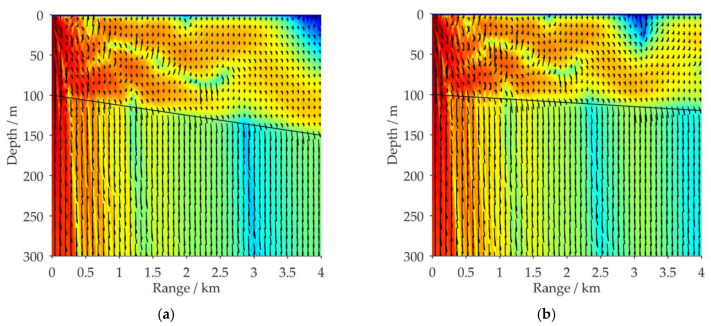

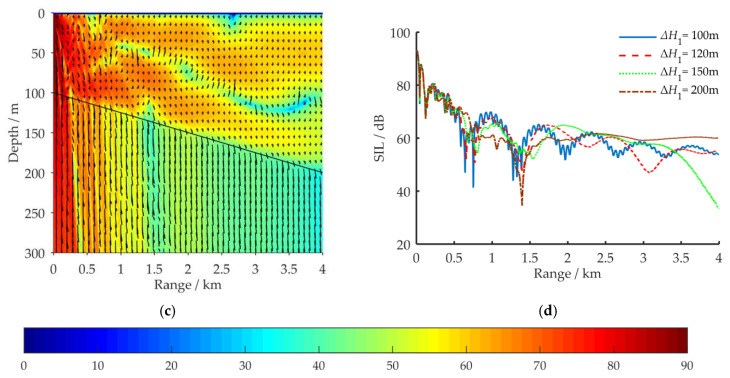
Distribution of the 100 Hz acoustic energy flux (SIL) in a full waveguide of shallow water with wedge-shaped downhill sea bottoms; aside from Δ*H*_1_, the environmental parameters are the same as in Figure 2a: (**a**) Δ*H*_1_ = 120 m; (**b**) Δ*H*_1_ = 150 m; (**c**) Δ*H*_1_ = 200 m; (**d**) comparison results of SIL curves for four simulation conditions: Δ*H*_1_ = 100, 120, 150, and 200 m, with a receiving depth of *z* = 50 m.

Like Figure 5, Figure 7 illustrates the distribution of the 100 Hz acoustic energy flux in a full waveguide of shallow water with a wedge-shaped downhill sea bottom. Aside from Δ*H*_1_, the environmental parameters are the same as in Figure 2a. Figure 7a–c respectively depict the simulation results for Δ*H*_1_ = 120, 150, and 200 m with corresponding inclinations of α_2_ = 0.29°, 0.72°, and 1.43°. Figure 7d shows a comparison of the propagation characteristics of the 100 Hz acoustic energy flux for four sea bottom simulation conditions: Δ*H*_1_ = 100, 120, 150, and 200 m. The receiving depth in the sea water is *z* = 50 m.

**Figure 8:**
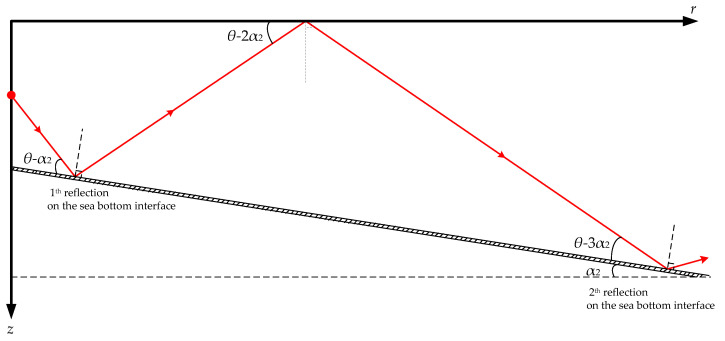
Distribution of the ray tracing when acoustic signals propagate in a shallow water environment with wedge-shaped downhill sea bottom, and the downhill slope angle of the wedge-shaped bottom is α_2_.

By comparing the simulation results in Figure 4 and Figure 7, it can be seen that under the influence of the downhill slope angle of the sea bottom *α*_2_, after its *n*th reflection on the sea bottom interface, the grazing angle of each excited normal mode is decreased by (2*n-*1)*α*_2_ degrees, as shown in Figure 8. This is the opposite influence of that observed for the wedge-shaped uphill sea bottom. For VLF sound propagation in shallow water with a wedge-shaped downhill sea bottom, the propagation characteristics of the high-order normal modes, which correspond to large grazing angles, are continuously coupled to the low-order normal modes, which correspond to small grazing angles, significantly weakening the leakage effect of the acoustic energy to the sea bottom. The larger the grazing angle α_2_ is, the more high-order normal modes are coupled to the low orders, and the less acoustic energy leaks into the sea bottom. The wedge-shaped downhill sea bottom would have an enhancement effect on the propagation of VLF signals in shallow water.

### 3.3. The Effects of Geoacoustic Parameters on the Characteristics of VLF Sound Propagation

In this section, we discuss the effects of three geoacoustic parameters, the density *ρ_b_* of the sea bottom, P-wave speed *c_p_*, and S-wave speed *c_s_* in the sea bottom, on VLF sound propagation in full shallow water waveguides. The effect laws of the above three parameters are respectively discussed in Section 3.3.1, Section 3.3.2 and Section 3.3.3

#### 3.3.1. Effect of the Density of the Sea Bottom on the Characteristics of VLF Sound Propagation

**Figure 9:**
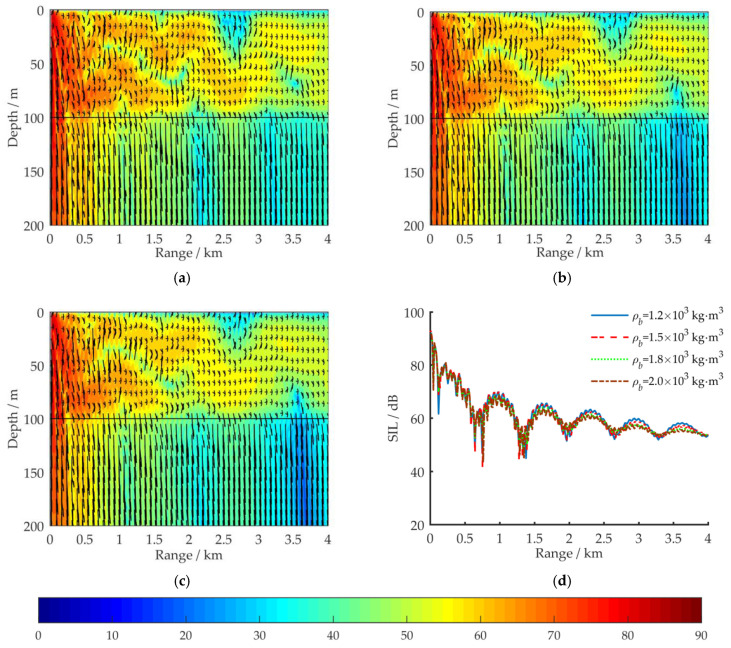
Distribution of the 100 Hz acoustic energy flux (SIL) in a full waveguide of shallow water for different sea bottom densities: (**a**) *ρ_b_* = 1.2 × 10^3^ kg/m^3^; (**b**) *ρ_b_* = 1.8 × 10^3^ kg/m^3^; (**c**) *ρ_b_* = 2.0 × 10^3^ kg/m^3^; (**d**) comparison results of SIL curves for four types of simulation conditions: *ρ_b_* = 1.2 × 10^3^, 1.5 × 10^3^, 1.8 × 10^3^, and 2.0 × 10^3^ kg/m^3^, with a receiving depth of *z* = 50 m.

Figure 9a–c demonstrates the propagation characteristics of the acoustic energy flux for different sea bottom densities under the simulation conditions shown in Figure 2a. The sea bottom densities in Figure 9a–c are *ρ_b_* = 1.2 × 10^3^, 1.8 × 10^3^, and *ρ_b_* = 2.0 × 10^3^ kg/m^3^, respectively. The remaining parameters of the shallow water environment are the same as in Figure 2a.

By comparison ofFigure 2a and Figure 9, it can be seen that, as the density of the sea bottom increases, the acoustic energy flux in the sea water decreases and more acoustic energy is transmitted into the sea bottom. These phenomena become more pronounced as the propagation range of the simulation increases, and based on the reflection rules for fluid/elastic interfaces [28], they can be further analyzed as follows.

The reflection coefficient *R* of the fluid/solid interface is given by Equation (15):

(15) $$R=\frac{Z_{p}\cos^{2}2\theta_{pt}+Z_{s}\sin^{2}2\theta_{st}-Z}{Z_{p}\cos^{2}2\theta_{pt}+Z_{s}\sin^{2}2\theta_{st}+Z}$$

$$Z=\frac{\rho_{1}c_{1}}{\sin\theta }\;Z_{p}=\frac{\rho_{b}c_{p}}{\cos\theta_{pt}}\;Z_{s}=\frac{\rho_{b}c_{s}}{\cos\theta_{st}}$$

where *ρ*_1_*c*_1_ is the acoustic wave impedance in sea water, *ρ_b_c_p_* and *ρ_b_c_s_* are respectively the P-wave impedance and S-wave impedance at the sea bottom, *θ* is the grazing angle when an acoustic wave is incident on the fluid/elastic interface, and *θ_pt_* and *θ_st_* are respectively the refraction angles of the P-wave and S-wave in the elastic medium below the interface. The three sets of parameters (*c*_1_, *θ*), (*c_p_*, *θ_pt_*)*,* and *(c_s_*, *θ_st_*) satisfy Snell’s law [28].

Under the simulation conditions shown in Figure 2a and Figure 9, the sound speed on sides of the water/bottom interface satisfies the relationship *c_p_* > *c*_1_ > *c_s_*. Under this premise, there is a critical grazing angle *θ*′ that satisfies the relationship sin*θ_pt_*′ = *c_p_*/*c*_1_·cos*θ*′ = 1. When an acoustic wave is incident on the interface at a grazing angle larger than *θ*′, at the sea bottom the P-wave, which is formed by the acoustic wave refraction, propagates as a nonuniform wave, and it cannot carry acoustic energy into the sea bottom. However, at the sea bottom the S-wave, which is formed by the acoustic wave, always propagates normally, and it still carries acoustic energy into the sea bottom. As the S-wave impedance increases, more acoustic energy is carried from the sea water to the sea bottom, and the modulus of the reflection coefficient *R* decreases. Therefore, when the sea bottom density increases, acoustic energy is more easily transmitted into the sea bottom via propagation, leading to the attenuation of the SIL in propagation. A comparison of Figure 2a and Figure 9 reveals that they are consistent with this analysis.

#### 3.3.2. Effect of the P-Wave Speed in the Sea Bottom on the Characteristics of VLF Sound Propagation

**Figure 10:**
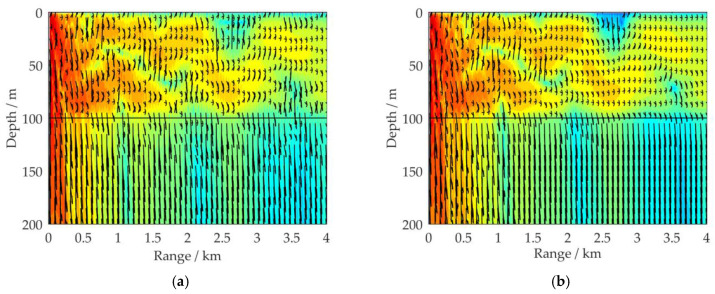

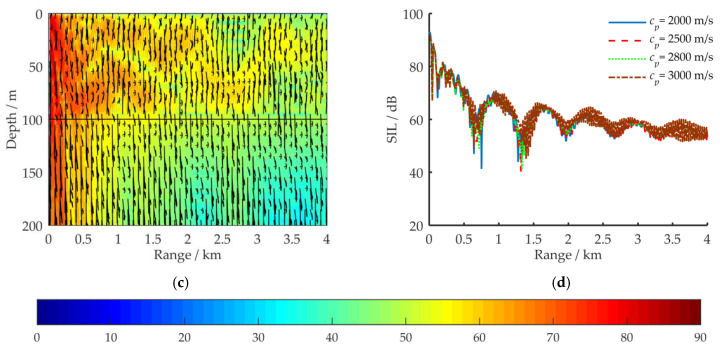
Distribution of the 100 Hz acoustic energy flux (SIL) in a full waveguide of shallow water for different P-wave speed in sea bottom. (**a**) *c_p_* = 2500 m/s; (**b**) *c_p_* = 2800 m/s; (**c**) *c_p_* = 3000 m/s; (**d**) comparison results of SIL curves for four types of simulation conditions: *c_p_* = 2000, 2500, 2800, and 3000 m/s, with a receiving depth of *z* = 50 m.

Figure 10a–c demonstrates the propagation characteristics of acoustic energy flux for P-wave speeds of *c_p_* = 2500, 2800, and 3000 m/s, respectively, under the simulation conditions in Figure 2a. The remaining parameters of the shallow water environment are the same as in Figure 2a. By comparing Figure 2a and Figure 10, it can be seen that, as the P-wave speed in the sea bottom increases, the acoustic energy flux in the sea water increases and the interference structure of the SIL becomes more complex. Using Equation (15) and Snell’s Law, these simulation results can be further analyzed as follows.

As the P-wave speed *c_p_* in the sea bottom increases, the modulus of *R* increases and the critical grazing angle *θ*′ increases, which shortens the horizontal span of the acoustic energy in the sea water. Therefore, more acoustic energy is reflected into the sea water rather than transmitted to the sea bottom as *c_p_* increases, leading to an increase of the SIL in propagation. The comparison between Figure 2a and Figure 10 is consistent with this analysis.

#### 3.3.3. Effect of S-Wave Speed in the Sea Bottom on the Characteristics of VLF Sound Propagation

**Figure 11:**
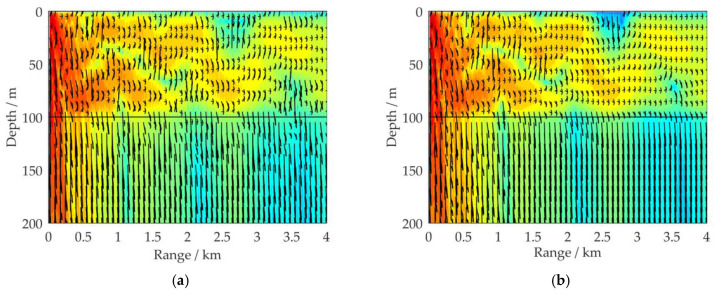

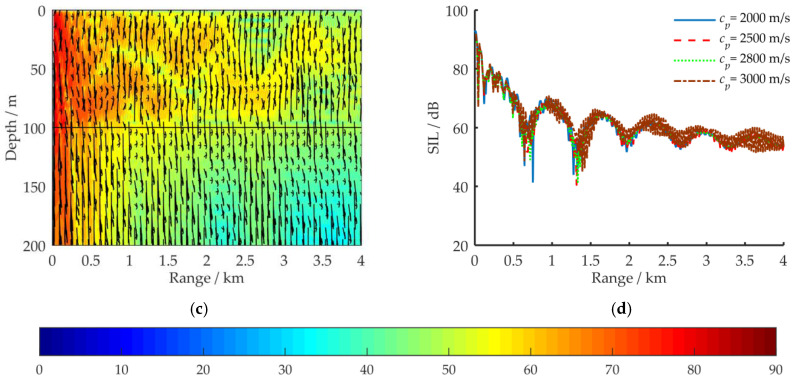
Distribution of the 100 Hz acoustic energy flux (SIL) in a full waveguide of shallow water for different S-wave speeds in sea bottom. (**a**) *c_s_* = 400 m/s; (**b**) *c_s_* = 600 m/s; (**c**) *c_s_* = 800 m/s; (**d**) comparison results of SIL curves for four types of simulation conditions: *c_p_* = 400, 600, 800, and 1000 m/s, with a receiving depth of *z* = 50 m.

Figure 11a–c demonstrates the propagation characteristics of the acoustic energy flux for S-wave speeds of *c_s_* = 400, 600, and 800 m/s, respectively, under the simulation condition in Figure 2a. The remaining parameters of the shallow water environment are the same as in Figure 2a.

Under the premise that *c*_1_ > *c_s_*, the S-wave formed by the acoustic wave refraction can propagate to the sea bottom and can carry some acoustic energy to the bottom. The greater the speed of the S-wave is, the more the acoustic energy in the sea water is able to penetrate into the sea bottom and the greater the transmission loss of the acoustic energy in the seawater.

## 4. Conclusions

This paper demonstrates the power of FEM for handling VLF sound propagation in a full waveguide of shallow water. By taking the acoustic energy flux as the research object and implementing FEM, the effects of the sea bottom topography and the geoacoustic parameters on the characteristics of VLF sound propagation and its corresponding mechanisms are investigated through numerical simulation and acoustic theory. The research results provide a theoretical reference for the research, development, and application of underwater acoustic engineering equipment for VLF sound in shallow water, and also can be used to develop new sensors and equipment which unify underwater acoustic signals and the seismic acoustic signals in underwater detection.

From this study, we draw the following specific conclusions:

(1) According to the simulation results, FEM is highly applicable for the calculation of sound propagation in complex sea environments, especially for sound propagation in range-dependent full shallow water waveguides. Our results indicate that the underwater acoustic field prediction simulated by FEM is consistent with the predictions obtained by other shallow water simulation methods.
(2) Both wedge-shaped uphill and downhill sea bottoms can significantly influence VLF sound propagation in shallow water. Compared with a horizontal sea bottom, under the influence of the uphill slope angle α_1_, the low-order normal modes, which correspond to small grazing angles, are continuously coupled to the high-order normal modes, which correspond to large grazing angles, significantly strengthening the leakage effect of the acoustic energy to the sea bottom and relieving the fluctuation. The larger the slope angle α_1_ is, the more the low-order normal modes are coupled to the higher order ones, the more the acoustic energy leaks into the sea bottom, the faster the acoustic energy attenuates in the sea water, the fewer the normal mode orders, and the simpler the normal mode interference in far-field regions. The influence of a wedge-shaped downhill sea bottom is exactly the opposite. Under the influence of the downhill slope angleα_2_, the high-order normal modes are continuously coupled to the low-order normal modes, and the leakage effect of the acoustic energy to the sea bottom is significantly weakened.
(3) By considering the sea bottom as an elastic medium, the effects of geoacoustic parameters of the sea bottom, namely, the P-wave speed *c_p_*, S-wave speed *c_s_*, and density *ρ_b_*, on VLF sound propagation in a full waveguide can be analyzed based on the reflection rules of the fluid/elastic interface. Under the condition *c_p_* > *c*_1_ > *c_s_*, as *c_p_* increases, the critical incidence angle on the fluid/sediment interface and the fluctuation cycle of the acoustic energy decrease, while the acoustic energy is more confined to propagating in the fluid layer. However, the effects of *c_s_* and *ρ_b_* are the opposite: increasing *c_s_* or *ρ_b_* causes more acoustic energy to be carried into the sea bottom, thereby increasing the attenuation rate of the acoustic energy in the fluid layer.

## Data Availability

No new data were created or analyzed in this study. Data sharing is not applicable to this article.
